# Dacomitinib overcomes acquired resistance to osimertinib in advanced NSCLC patients with EGFR L718Q mutation: A two-case report

**DOI:** 10.1097/MD.0000000000038789

**Published:** 2024-07-12

**Authors:** Jielin Li, Meizi Jin, Yuzhu Diao, Xiaoling Li

**Affiliations:** a Department of Thoracic Internal Medicine, Liaoning Cancer Hospital and Institute, Cancer Hospital of China Medical University, Shenyang, China.

**Keywords:** dacomitinib, EGFR L718Q mutation, NSCLC, osimertinib resistance

## Abstract

**Rationale:**

Acquired resistance still inevitably occurs in patients treated with third-generation TKI osimertinib. Although the EGFR L718Q mutation has been reported as a scarce mechanism of osimertinib resistance, advanced therapeutic strategies are still in development. In this report, we included 2 cases of patients who acquired EGFR L858R/L718Q mutation after osimertinib and were overcome by dacomitinib.

**Patient concerns:**

Case 1: A 77-year-old woman was diagnosed with stage IV lung adenocarcinoma. Case 2: A 64-year-old woman was diagnosed with stage IV lung adenocarcinoma.

**Diagnoses:**

Case 1: The patient was diagnosed with adenocarcinoma with EGFR L858R mutation. Since then, treatment with gefitinib was administrated, leading to a progression-free survival of 18 months. The treatment was switched to osimertinib based on the detection of EGFR T790M mutation, resulting in a progression-free survival of 24 months. Subsequently, EGFR L718Q mutation was detected. Case 2: The patient was diagnosed with adenocarcinoma with EGFR L858R mutation. Icotinib was used as the first-line treatment for 7 months. Osimertinib was applied as the second-line treatment for 13 months based on the EGFR T790M mutation. Subsequently, EGFR L718Q mutation was detected.

**Interventions:**

Case 1: Dacomitinib was administered. Case 2: Dacomitinib was administered.

**Outcomes:**

Case 1:The progression-free survival was 8 months. Case 2: The progression-free survival was 3 months.

**Lessons:**

Dacomitinib is a potential treatment option for NSCLC patients with EGFR L718Q mutation after resistance to Osimertinib. Further research is needed to validate the efficacy of Dacomitinib in this context.

## 1. Introduction

The incidence and mortality of lung cancer rank first in the world for malignant tumors.^[1]^ The latest statistics showed that the incidence of lung cancer in China not only ranks first among all cancers but also continue growing.^[2]^ Lung cancer can be catergorized into small cell lung cancer (SCLC) and non-small cell lung cancer (NSCLC), with NSCLC comprising 80% to 85% of cases. Epidermal growth factor receptor (EGFR) gene mutations are commonly found in NSCLC patients and serve as therapeutic targets. Several large studies demonstrated that epidermal growth factor receptor tyrosine kinase inhibitors (EGFR-TKIs) were superior to cytotoxic chemotherapy in patients with EGFR mutations and have significant advantages in terms of objective response rate and progression-free survival (PFS).^[3–7]^ However, acquired resistance remains inevitable within patients treated with the third generation tyrosine kinase inhibitor (TKI) osimertinib. The EGFR L718Q mutation is an infrequent mechanism of osimertinib resistance and its role in therapeutic application requires further investigation.^[8]^ Here, we report 2 cases of advanced NSCLC patients with concomitant EGFR L858R/L718Q mutations after osimertinib resistance. In both cases, additional treatment with dacomitinib showed significant therapeutic effects, suggesting that dacomitinib could be a novel treatment method for NSCLC patients with EGFR L718Q mutation.

## 2. Case presentation

### 2.1. Case 1

**Figure 1.**
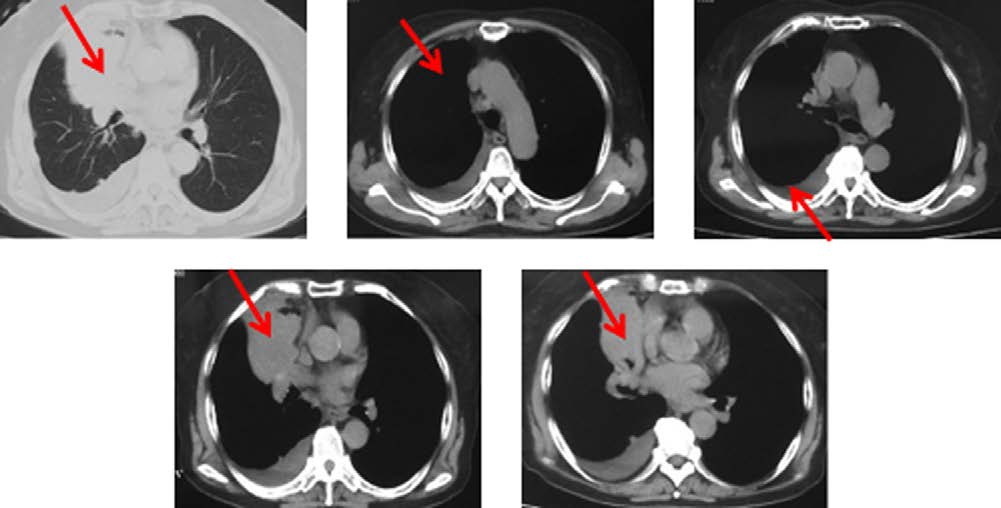
The chest CT scan demonstrated right middle lobe mass, right hilum and mediastinal lymph node metastasis, right pleural effusion at baseline. CT=computed tomography.

In October 2016, a 77-year-old woman was admitted to Canadian Centenary Health Center with main complaints of cough and blood in sputum. She was diagnosed with hypertension for more than 10 years and the situation was well controlled by oral medications. There was no history of smoking and drinking and no family history of cancer. Physical examination revealed weakened breath sound and dull percussion in right lung. A computed tomography (CT) showed a mass in right middle, right pleural effusion, right hilar and mediastinal lymphadenectasis, with no metastases in the abdomen and bone and no evidence of intracranial metastases (Fig. 1). Thoracentesis was performed in October 2016. Pathology reported metastatic adenocarcinoma of the lung. The immunohistochemistry was displayed as follows: CK7(+), TTF-1(+), CK20(-), CDX2(-), GCDFP(-), mammaglobin(-), ER(-), PR(-). The gene detection showed EGFR L858R mutation in exon 21. This patient was diagnosed with lung adenocarcinoma (cT4N2M1a, stage IV).

**Figure 2.**
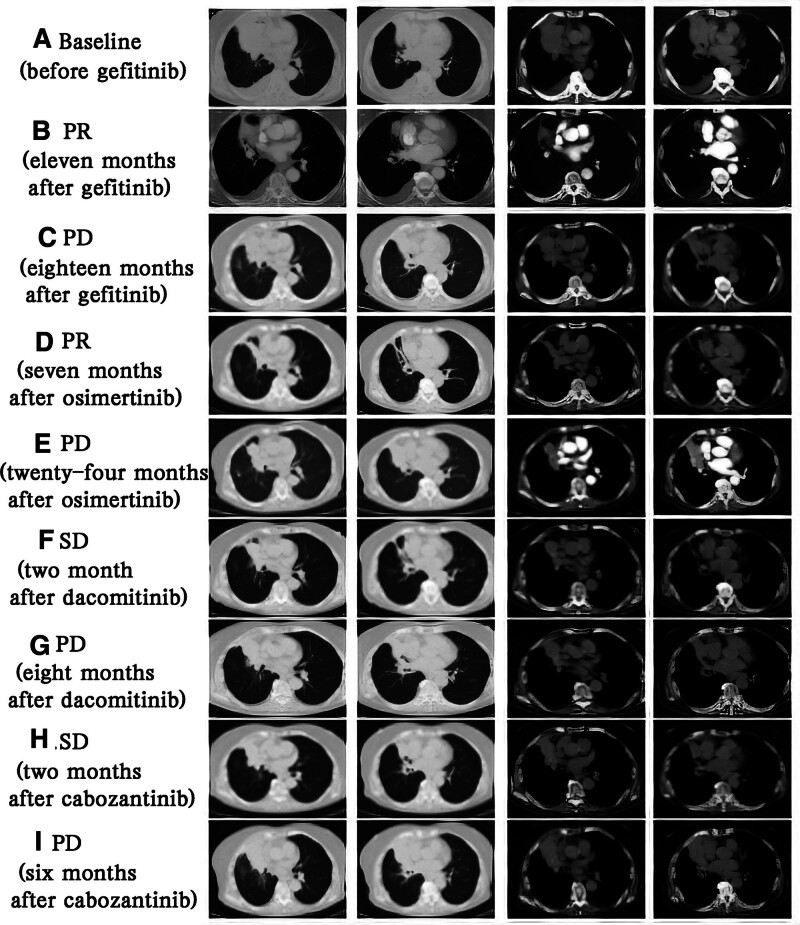
(A) Primary lung tumor at baseline before gefitinib. (B) PR after 11 months of gefitinib. (C) PD after 18 months of gefitinib. (D) PR after 7 months of osimertinib. (E) PD after 24 months of osimertinib. (F) SD after 2 months of dacomitinib. (G) PD after 8 months of dacomitinib. (H) SD after 2 months of cabozantinib. (I) PD after 6 months of cabozantinib. PD = progressive disease, PR = partial response, SD = sable disease.

**Figure 3.**
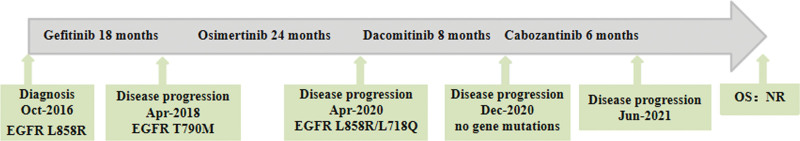
Treatment timeline (Case 1).

Starting from October 2016, the patient was treated with gefitinib (250 mg qd orally). A partial response was achieved after 11 months (Fig. 2A and B). In April 2018, she came to Liaoning Cancer Hospital with chest tightness, shortness of breath, and large pleural effusion in right lung confirmed by ultrasonography. Thoracentesis was then performed, and tumor was identified in pleural effusion. A chest CT showed enlarged right lung mass with lung and pleura metastasis (Fig. 2C). Liquid biopsy using plasma and pleural effusion was performed. Next-generation sequencing (NGS) reported EGFR L858R mutation in exon 21 (frequency: 10%), and EGFR T790M mutation in exon 20 (frequency: 2.7%) in pleural effusion, but not in plasma. For further treatment, osimertinib was administered (80 mg qd orally) starting from April 2018. The disease condition was well controlled, and a partial response was identified after 7 months (Fig. 2D). Two years later, in April 2020, this patient developed low back pain, bone metastasis (T8 and L3) confirmed by positron emission tomography-computed tomography. Concomitantly, a chest CT indicated enlarged right lung mass and mediastinal lymphadenopathy (Fig. 2E). NGS of plasma revealed EGFR L858R mutation in exon 21 (frequency: 4.4%) and a new EGFR L718Q mutation in exon 18 (frequency: 2.1%). In response, dacomitinib was administrated (30 mg qd orally) since April 2020 along with bone radiotherapy. A stable disease stage was achieved after 2 months, with the tumor shrinking while stabilizing (Fig. 2F). She tolerated the treatment well with grade 1 skin rash and paronychia. Progressive disease was identified again after 8 months (Fig. 2G). There was no additional gene mutation detected by NGS of plasma. Then cabozantinib was used by the patient herself (40 mg qd orally) starting in December 2020. A sable disease was achieved after 2 months (Fig. 2H). Adverse reactions included high blood pressure, skin rash, bleeding gums, loss of appetite and so on, leading to a poor tolerance of cabozantinib treatment. In June 2021, she complained cough and blood in sputum, and a chest CT showed an enlarged right lung lesion (Fig. 2I). As of now, the overall survival has not been reached. The treatment timeline is shown in Figure 3.

### 2.2. Case 2

**Figure 4.**
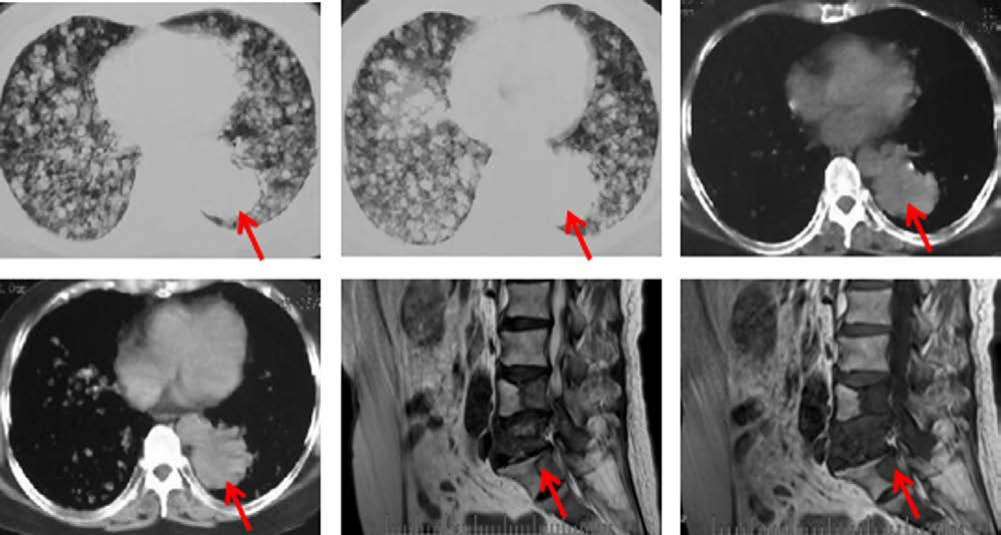
The chest CT scan demonstrated left lower lobe mass and double lung diffuse multiple metastasis, the MRI scan demonstrated bone metastasis at baseline. CT=computed tomography, MRI=magnetic resonance imaging.

A 64-year-old woman was admitted to Liaoning Cancer Hospital in August 2019 with main complaints of cough, blood in sputum and low back pain. There is no history of smoking, drinking, medical history and family history of cancer in patient’s self-report. Also, there is no significant abnormalities on physical examination. A CT scan showed a mass in left lower lobe, bilateral lung metastases and no metastases in the abdomen or brain. The magnetic resonance imaging indicated bone metastasis (Fig. 4). Bronchoscopy was performed in August 2019 and pathology reported adenocarcinoma. The EGFR L858R mutation in exon 21 (frequency:88.6%) and TP53 mutation in exon 10 (frequency:46.5%) were detected by NGS. The patient was diagnosed with lung adenocarcinoma (cT4N0M1c, stage IV).

**Figure 5.**
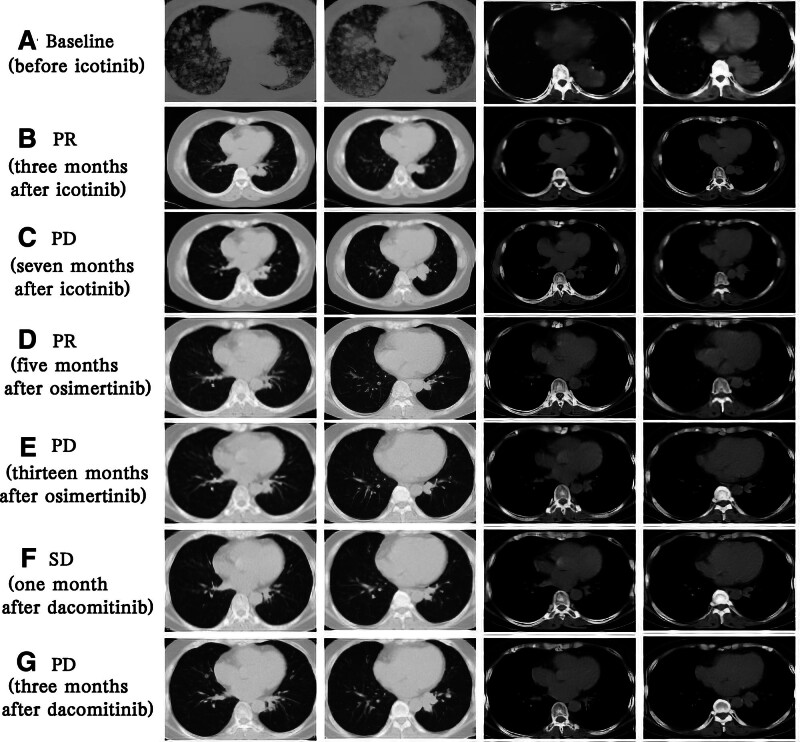
(A) Primary lung tumor, double lung metastasis at baseline before icotinib. (B) PR after 3 months of icotinib. (C) PD after 7 months of icotinib. (D) PR after 5 months of osimertinib. (E) PD after 13 months of osimertinib. (F) SD after 1 month of dacomitinib. (G) PD after 3 months of dacomitinib. PD = progressive disease, PR = partial response, SD = sable disease.

**Figure 6.**
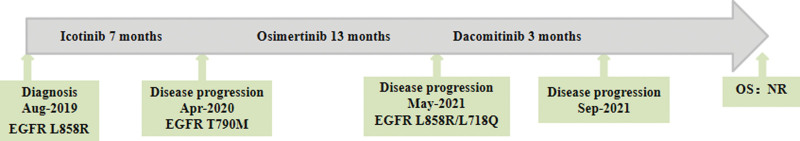
Treatment timeline (Case 2).

Since September 2019, the patient was treated with icotinib (125 mg tid orally). A partial response was achieved after 3 month (Fig. 5A and B). In April 2020, a chest CT showed that the tumor in the left lower lobe was enlarged (Fig. 5C). The EGFR L858R mutation in exon 21 (frequency: 0.8%) and T790M mutation in exon 20 (frequency: 0.2%) were detected by NGS of plasma. Osimertinib was administered (80 mg qd orally) starting from April 2020. The disease was well controlled, and a partial response was observed after 5 month (Fig. 5D). In May 2021, based on the chest CT results, the patient developed disease progression (Fig. 5E). The EGFR L858R mutation in exon 21 (frequency: 1.1%) and TP53 mutation in exon 10 (frequency: 0.2%) and a new EGFR L718Q mutation in exon 18 (frequency: 0.9%) were identified by NGS of plasma. Dacomitinib was challenged (30 mg qd orally) since June 2021. A stable disease condition was achieved after 1 month (Fig. 5F). The patient tolerated well and no significant adverse reactions. In September 2021, her disease had progressed again (Fig. 5G). At present, the overall survival has not reached. The treatment timeline is shown in Figure 6.

### 2.3. Ethical conduct statement

This study was conducted in compliance with the Declaration of Helsinki. The design of the study was approved by the appropriate Ethics Review Board of Liaoning Cancer Hospital and Institute (No.: 20211171), and the patient provided informed consent for publication of this report.

## 3. Discussion and conclusions

Lung cancer is the most common malignant tumor in clinical practice. Several studies have confirmed that EGFR-TKIs have become standard medicines for the first-line treatment of NSCLC with EGFR gene sensitive mutations. However, as reported, most patients would inevitably develop acquired drug resistance after 9 to 15 months of treatment with the first-generation EGFR-TKIs.^[9]^ Among the reasons responsible for acquired drug resistance development, secondary mutations are the main reason, in which, the T790M mutation in exon 20 accounts for about 50% of patients with secondary drug resistance.^[10]^ The results of the AURA phase III trial showed that osimertinib compared with traditional platinum-containing dual-drug chemotherapy in patients with T790M mutation, documented a objective response rate of 71% versus 31%, PFS of 11.0 months versus 4.2 months and overall survival (OS) of 26.8 months versus 22.5 months.^[7–11]^ The consequent FLAURA phase III trial confirmed that Osimertinib, when used as the first line treatment for locally advanced or metastatic EGFR-mutant NSCLC, significantly prolonged the PFS (from 10.2 months to 18.9 months) and OS (from 31.8 months to 38.6 months) compared with standard first-generation EGFR-TKIs (gefitinib, erlotinib).^[12,13]^ However, osimertinib resistance still develops roughly after 19 months for first-line treatment and 11 months for second-line treatment.^[14]^

In recent years, the resistance mechanism of third-generation EGFR-TKIs has been demonstrated. The mechanisms can be roughly divided into 2 categories: EGFR-dependent resistance mechanisms and EGFR-independent resistance mechanisms.^[14,15]^ EGFR-dependent resistance mechanisms are usually associated with additional EGFR mutations, which disrupt the binding of osimertinib through changes in binding site by allosteric/conformational transitions.^[14]^ The most common resistance mutation of osimertinib is the C797S mutation. This point mutation in EGFR exon 20 disrupts the covalent binding of C797 and EGFR-TKIs, leading to development of osimertinib resistance.^[16–18]^ Other than C797S mutation, EGFR mutations including G796R/S/D mutation, L792X mutation, L718Q mutation, G719A mutation, G724S mutation and exon 20 insertion could also lead to development of osimertinib resistance.^[17–20]^ EGFR-independent mechanisms of resistance include 4 categories as follow: Bypass pathway activation: MET amplification, HER2 amplification; Activation of other bypass signaling pathways: cell-cycle gene alterations (CDKN2A E27fs mutation, CCND amplification, CDK4/6 amplification, CCNE1 amplification), oncogenic gene fusions (BRAF, FGFR3, NTRK1, RET, ALK, ROS1), insulin-like growth factor 1 (IGFR1) activation, fibroblast growth factor receptor (FGFR) amplification; Downstream pathway activation: PI3KCA mutation, RAS mutation, PTEN deletion; and Epithelial-to-mesenchymal transition and histologic transformation: small cell transformation or squamous cell transformation.^[17–19,21]^

Robichaux et al demonstrated that structure–function-based groups can identify classes of drugs that may be effective for entire groups of mutations, highlighting that mutations in different regions of the gene can induce similar changes in protein structure.^[20]^ Several studies have confirmed that L718Q mutation is one of the gene mutations that causes osimertinib resistance. The L718Q mutation affected the spatial conformation of the EGFR-osimertinib complex and reduced the covalent binding efficiency, thereby introducing drug resistance.^[22]^ In vitro studies confirmed that EGFR L858R/L718Q mutations were sensitive to afatinib and dacomitinib, but resistant to erlotinib and brigatinib.^[23]^ Case reports have showed that the patient harboring EGFR L858R/L718Q respond to afatinib, but not to dacomitinib.^[24–26]^ However, in our cases, according to the results of in vitro studies, we boldly attempted to use dacomitinib to treat patients with EGFR L858R/L718Q mutations, and proven effective. The PFS was prolonged for 8 months and 3 months respectively in 2 different cases, indicating that the second-generation TKI dacomitinib had potential therapeutic significance for acquired EGFR L718Q mutation and further clinical validation is required.

TP53 is a tumor suppressor gene encoding the tumor protein P53 and is one of the common mutated genes in NSCLC. The P53 regulates the cellular response to a variety of stress signals by inducing senescence, arresting cell cycle and/or apoptosis, thereby playing an important role of tumor suppressor protein.^[27]^ Previous studies reported that EGFR combined with TP53 mutations could change the sensitivity of tumor tissues to TKI and chemotherapy and the long-term prognosis of patients.^[28,29]^ In the present cases, Case 1 harbored only an EGFR mutation, while Case 2 had both EGFR and TP53 mutations. Therefore, the PFS of Case 2 was shorter than Case 1. This difference might be related to the presence of both EGFR and TP53 mutations.

## 4. Conclusion

In summary, this study provided evidence for efficacy of dacomitinib targeting concomitant EGFR L858R/L718Q mutations after osimertinib resistance, indicating that the second-generation EGFR-TKI could serve as a potential treatment option. Further, these therapeutic effects might be influenced by TP53. At the same time, cabozantinib could be considered as a treatment method for NSCLC patients without EGFR mutations. Further investigations are needed to explore more mechanisms and treatments for osimertinib resistance.

## Author contributions

**Conceptualization:** Jielin Li.

**Data curation:** Jielin Li, Meizi Jin, Yuzhu Diao.

**Writing – original draft:** Jielin Li.

**Writing – review & editing:** Xiaoling Li.
